# Supplementary material for: Small hydropower plants and livelihoods of the local population in rural Vietnam
Source: PLoS One. 2025 Mar 24;20(3):e0317247. doi: 10.1371/journal.pone.0317247 (PMC11932490; doi:10.1371/journal.pone.0317247)
Supplement: S1 Table — (DOCX) [file pone.0317247.s001.docx]

S 1 Table. Agricultural variables calculated for each household

| *Variable* | *Definition* |
| --- | --- |
| Agricultural Income | Total agricultural income in 2005 PPP USD |
| Cultivated Land | Total area of cultivated land in ha |
| Share of Irrigated Land | Share of total cultivated land that is irrigated |
| Agricultural Shocks | Number of agricultural shocks (floods, droughts, unusually heavy rainfall, crop and storage pests, livestock diseases, and landslide/erosion) a household is expecting in the upcoming year. The household was asked for each of the shocks summed up as “agricultural shocks” if they think they will occur and how often they think these shocks will occur. |
| Expected Number Droughts | The number of droughts a household is expecting in the coming year |
| No. Household Members work on-farm | The number of household members engaged in farming on the household’s own farm |
| No. Household Members work off-farm | The number of household members engaged in off-farm work such as non-farm owned businesses, casual and permanent off-farm labor |
