# Supplementary material for: Small hydropower plants and livelihoods of the local population in rural Vietnam
Source: PLoS One. 2025 Mar 24;20(3):e0317247. doi: 10.1371/journal.pone.0317247 (PMC11932490; doi:10.1371/journal.pone.0317247)
Supplement: S2 Table — (DOCX) [file pone.0317247.s002.docx]

S 2 Table. Fixed effects regression for the impact of HPPs using lagged values for control variables

|  | *Agricultural income* | *Cultivated*  *land* | *Share*  *irrigated land* | *Expected number*  *droughts* | *Poverty head-*  *count ratio* | *Gini*  *coefficient* |
| --- | --- | --- | --- | --- | --- | --- |
|  |  |  |  |  |  |  |
| *Panel A: Whole sample* |  |  |  |  |  |  |
| Distance to nearest HPP | -205.38*** | -0.03** | -0.004 | -0.04 | 0.009 | 0.001 |
|  | (48.67) | (0.01) | (0.004) | (0.03) | (0.005) | (0.004) |
| Distance to nearest HPP located upstream | -224.95 | -0.03 | -0.008 | -0.12*** | -0.01 | 0.007 |
|  | (160.31) | (0.08) | (0.01) | (0.04) | (0.007) | (0.005) |
| Distance to nearest HPP located downstream | 2,275.3 | -0.35 | -0.12 | 0.87 | 0.68** | 0.15 |
|  | (2,084.29) | (0.22) | (0.17) | (1.11) | (0.25) | (0.12) |
| Controls | yes | yes | yes | yes | yes | yes |
| *Panel B: Dak Lak* | | | | | | |
| Distance to nearest HPP | -211.64*** | -0.014 | -0.006 | -0.01 | 0.02*** | 0.005 |
|  | (53.15) | (0.013) | (0.006) | (0.1) | (0.004) | (0.003) |
| Distance to nearest HPP located upstream | -202.28*** | 0.09*** | -0.02*** | -0.09*** | 0.02* | 0.005 |
|  | (69.00) | (0.007) | (0.004) | (0.03) | (0.01) | (0.01) |
| Distance to nearest HPP located downstream | -2,282.6 | -0.35 | -0.12 | 0.85 | 0.68** | 0.15 |
|  | (2,091.85) | (0.22) | (0.17) | (1.12) | (0.25) | (0.12) |
| Controls | yes | yes | yes | yes | yes | yes |
| Standard errors clustered at village level in parentheses, ^*^ *p* < 0.1, ^**^ *p* < 0.05, ^***^ *p* < 0.01, Source: Own calculation from TVSEP data | | | | | | |
