# Supplementary material for: Small hydropower plants and livelihoods of the local population in rural Vietnam
Source: PLoS One. 2025 Mar 24;20(3):e0317247. doi: 10.1371/journal.pone.0317247 (PMC11932490; doi:10.1371/journal.pone.0317247)
Supplement: S3 Table — (DOCX) [file pone.0317247.s003.docx]

S 3 Table. Results for equations (1) & (2) using numbers of HPPs upstream and downstream

|  | *Agricultural. income* | *Cultivated*  *land* | *Share*  *irrigated land* | *Expected number*  *droughts* | *Poverty head-*  *count ratio* | *Gini*  *coefficient* | |
| --- | --- | --- | --- | --- | --- | --- | --- |
|  |  |  |  |  |  |  |  |
| *Panel A: Whole Sample* | | | | | | | |
| Number of HPP  located downstream | 106.3 | -0.08*** | -0.002 | 0.07*** | -0.001 | 0.003 | |
|  | (269.2) | (0.03) | (0.01) | (0.02) | (0.008) | (0.006) | |
| Number of HPP  located upstream | 299.42* | 0.006 | 0.01 | -0.1* | -0.02*** | 0.001 | |
|  | (163.5) | (0.03) | (0.01) | (0.05) | (0.007) | (0.005) | |
| *Panel B: Dak Lak* | | | | | | | |
| Number of HPP  located downstream | 126.36  (379.24) | -0.05  (0.03) | 0.03  (0.02) | 0.07**  (0.03) | -0.01  (0.008) | -0.001  (0.007) | |
| Number of HPP  located upstream | 338.6*  (173.0) | -0.022  (0.026) | 0.009  (0.012) | -0.04  (0.05) | -0.02***  (0.007) | 0.001  (0.005) | |
| Standard errors clustered at village level in parentheses, ^*^ *p* < 0.1, ^**^ *p* < 0.05, ^***^ *p* < 0.01, Source: Own calculation from TVSEP data | | | | | | |  |
