# Supplementary material for: Small hydropower plants and livelihoods of the local population in rural Vietnam
Source: PLoS One. 2025 Mar 24;20(3):e0317247. doi: 10.1371/journal.pone.0317247 (PMC11932490; doi:10.1371/journal.pone.0317247)
Supplement: S4 Table — (DOCX) [file pone.0317247.s004.docx]

S 4 Table. HPPs’ effect on cash crop production

|  | *Distance to nearest* | *Distance to nearest HPP* | *Distance to nearest HPP* |
| --- | --- | --- | --- |
|  | *HPP* | *downstream* | *upstream* |
| *Panel A: Whole sample* |  |  |  |
| Total cash crop production (kg) | -85.03* | 48,155 | -13.38 |
|  | (50.46) | (40,871) | (92.78) |
| *Panel B: Dak Lak* |  |  |  |
| Total cash crop production (kg) | -78.23 | 48,125 | 9.89 |
|  | (62.99) | (40,846) | (99.85) |
| Standard errors clustered at village level in parentheses, ^*^ *p* < 0.1, ^**^ *p* < 0.05, ^***^ *p* < 0.01, Source: Own calculation from TVSEP data | | | |
