# Supplementary material for: Small hydropower plants and livelihoods of the local population in rural Vietnam
Source: PLoS One. 2025 Mar 24;20(3):e0317247. doi: 10.1371/journal.pone.0317247 (PMC11932490; doi:10.1371/journal.pone.0317247)
Supplement: S5 Table — (DOCX) [file pone.0317247.s005.docx]

S 5 Table. Distance to nearest HPP with cash crop dummy as additional control variable

|  | *Agricultural income* | *Cultivated*  *land* | *Share*  *irrigated land* | *Expected Number*  *Droughts* | *Poverty head-*  *count Ratio* | *Gini*  *Coefficient* | |
| --- | --- | --- | --- | --- | --- | --- | --- |
|  |  |  |  |  |  |  |  |
| *Panel A: Whole sample* | | | | | | | |
| Distance to nearest HPP | **-**8.2 | **-**0.025*** | -0.004** | -0.004 | 0.003 | 0.0026* | |
|  | (19.36) | (0.008) | (0.002) | (0.013) | (0.002) | (0.001) | |
| Cash crop (yes/no) | 312.92* | 0.02 | 0.063*** | -0.084 | 0.026* | 0.017* | |
|  | (174.15) | (0.028) | (0.014) | (0.058) | (0.014) | (0.009) | |
| Distance to nearest HPP downstream | 1,917.63 | 0.042 | 0.072 | 0.871* | 0.139 | 0.128* | |
|  | (4,224.33) | (0.252) | (0.118) | (0.443) | (0.11) | (0.067) | |
| Cash crop (yes/no) | 171.48 | 0.065 | 0.127*** | -0.065 | 0.0001 | 0.018 | |
|  | (419.97) | (0.052) | (0.031) | (0.111) | (0.03) | (0.018) | |
| Distance to nearest HPP upstream | -32.07 | **-**0.009 | -0.013*** | **-**0.007 | **-**0.007 | 0.005 | |
|  | (42.7) | (0.021) | (0.003) | (0.035) | (0.005) | (0.004) | |
| Cash crop (yes/no) | 60.29 | -0.031 | 0.041** | -0.023 | 0.037* | 0.01 | |
|  | (221.17) | (0.032) | (0.016) | (0.082) | (0.021) | (0.015) | |
| *Panel B: Dak Lak* |  |  |  |  |  |  | |
| Distance to nearest HPP | -68.22* | -0.024 | -0.002 | 0.023 | 0.004 | 0.003 | |
|  | (34.95) | (0.016) | (0.004) | (0.016) | (0.005) | (0.002) | |
| Cash crop (yes/no) | 721.61** | 0.109** | 0.142*** | -0.126 | -0.003 | 0.011 | |
|  | (301.28) | (0.049) | (0.021) | (0.079) | (0.02) | (0.013) | |
| Distance to nearest | -1,900.1 | 0.046 | 0.074 | 0.871* | 0.137 | 0.132** | |
| HPP downstream | (4,241.79) | (0.252) | (0.117) | (0.444) | (0.109) | (0.066) | |
| Cash crop (yes/no) | 189.46 | 0.071 | 0.132*** | -0.063 | -0.001 | 0.018 | |
|  | (443.23) | (0.054) | (0.032) | (0.115) | (0.031) | (0.019) | |
| Distance to nearest | -69.65* | 0.013* | -0.011*** | 0.029* | -0.0002 | -0.003 | |
| HPP upstream | (35.47) | (0.007) | (0.003) | (0.016) | (0.005) | (0.003) | |
| Cash crop (yes/no) | 652.56 | 0.043 | 0.114*** | -0.067 | 0.005 | 0.019 | |
|  | (419.4) | (0.083) | (0.032) | (0.141) | (0.035) | (0.024) | |
| Standard errors clustered at village level in parentheses, ^*^ *p* < 0.1, ^**^ *p* < 0.05, ^***^ *p* < 0.01, Source: Own calculation from TVSEP data | | | | | | |  |
