# Supplementary material for: Small hydropower plants and livelihoods of the local population in rural Vietnam
Source: PLoS One. 2025 Mar 24;20(3):e0317247. doi: 10.1371/journal.pone.0317247 (PMC11932490; doi:10.1371/journal.pone.0317247)
Supplement: S6 Table — (DOCX) [file pone.0317247.s006.docx]

S 6 Table. Distance to nearest HPP with cash crop dummy and interaction terms as additional control variable

|  | *Agricultural income* | *Cultivated*  *land* | *Share irrigated*  *land* | *Expected number*  *droughts* | *Poverty head-*  *count ratio* | *Gini*  *coefficient* |
| --- | --- | --- | --- | --- | --- | --- |
|  |  |  |  |  |  |  |
| *Panel A: Whole Sample* |  |  |  |  |  |  |
| Interaction term HPP distance and cash crop | -21.9 | **-**0.008*** | **-**0.005*** | 0.003 | **-**0.0004 | **-**0.0004 |
|  | (15.5) | (0.003) | (0.001) | (0.004) | (0.001) | (0.0009) |
| Distance to nearest HPP | -5.48 | **-**0.024*** | -0.003* | -0.005 | 0.0035 | 0.003* |
|  | (19.22) | (0.008) | (0.002) | (0.013) | (0.002) | (0.001) |
| Cash crop (yes/no) | 690.45** | 0.15** | 0.148*** | **-**0.139 | 0.033 | 0.023 |
|  | (339.88) | (0.057) | (0.025) | (0.103) | (0.026) | (0.017) |
| Interaction term HPP distance downstr. and cash crop | -23.21 | -0.002 | -0.001 | **-**0.007 | -0.0004 | 0.001 |
|  | (33.95) | (0.002) | (0.001) | (0.006) | (0.001) | (0.001) |
| Distance to nearest HPP downstream | -1,985.91 | 0.037 | 0.071 | 0.865* | 0.137 | 0.13* |
|  | (4,231.65) | (0.25) | (0.117) | (0.45) | (0.111) | (0.067) |
| Cash crop (yes/no) | 391.27 | 0.086 | 0.134*** | -0.016 | 0.004 | 0.014 |
|  | (494.56) | (0.056) | (0.035) | (0.119) | (0.033) | (0.021) |
| Interaction term HPP distance upstream and cash crop | -42.45** | -0.004 | -0.004*** | 0.005 | 0.001 | **-**0.003** |
|  | (17.08) | (0.003) | (0.013) | (0.006) | (0.002) | (0.001) |
| Distance to nearest HPP upstream | -5.56 | **-**0.007 | -0.01*** | **-**0.01 | **-**0.007 | 0.006 |
|  | (43.64) | (0.021) | (0.003) | (0.035) | (0.006) | (0.004) |
| Cash crop (yes/no) | 772.49** | 0.037 | 0.112*** | -0.102 | 0.015 | 0.054** |
|  | (361.8) | (0.071) | (0.031) | (0.146) | (0.036) | (0.024) |
| *Panel B: Dak Lak* |  |  |  |  |  |  |
| Interaction term HPP distance and cash crop | 24.69 | **-**0.005 | **-**0.001 | 0.002 | **-**0.001 | 0.001 |
|  | (36.81) | (0.0075) | (0.002) | (0.007) | (0.002) | (0.001) |
| Distance to nearest HPP | -70.52* | **-**0.023 | -0.002 | 0.023 | 0.004 | 0.003 |
|  | (35.82) | (0.016) | (0.004) | (0.016) | (0.005) | (0.002) |
| Cash crop (yes/no) | 464.89 | 0.163* | 0.153*** | **-**0.148 | 0.011 | 0.001 |
|  | (468.67) | (0.084) | (0.031) | (0.12) | (0.029) | (0.019) |
| Interaction term HPP distance downstr. and cash crop | -23.46 | -0.002 | -0.001 | **-**0.005 | -0.0005 | 0.001 |
|  | (35.35) | (0.002) | (0.001) | (0.006) | (0.001) | (0.001) |
| Distance to nearest HPP downstream | -1,973.02 | 0.041 | 0.073 | 0.865* | 0.135 | 0.134** |
|  | (4,248.68) | (0.252) | (0.116) | (0.45) | (0.11) | (0.066) |
| Cash crop (yes/no) | 394.93 | 0.089 | 0.138*** | -0.026 | 0.003 | 0.013 |
|  | (500.51) | (0.057) | (0.036) | (0.121) | (0.034) | (0.02) |
| Interaction term HPP distance upstream and cash crop | -220.69 | 0.004 | -0.003 | **-**0.0003 | 0.003 | 0.004 |
|  | (143.98) | (0.016) | (0.007) | (0.025) | (0.006) | (0.005) |
| Distance to nearest HPP upstream | 83.33 | 0.011 | -0.009* | 0.029 | **-**0.001 | **-**0.001 |
|  | (97.81) | (0.012) | (0.005) | (0.024) | (0.006) | (0.003) |
| Cash crop (yes/no) | 1,576.4** | 0.024 | 0.127** | -0.066 | **-**0.008 | 0.036 |
|  | (698.12) | (0.103) | (0.051) | (0.207) | (0.043) | (0.031) |
| Standard errors clustered at village level in parentheses, ^*^ *p* < 0.1, ^**^ *p* < 0.05, ^***^ *p* < 0.01, Source: Own calculation from TVSEP data | | | | | | |
