# Supplementary material for: Small hydropower plants and livelihoods of the local population in rural Vietnam
Source: PLoS One. 2025 Mar 24;20(3):e0317247. doi: 10.1371/journal.pone.0317247 (PMC11932490; doi:10.1371/journal.pone.0317247)
Supplement: S8 Table — (DOCX) [file pone.0317247.s008.docx]

S 8 Table. Test for differences in means for households who dropped out and stayed in the sample

|  | *Mean for households who dropped out of the sample* | *Mean for households who stayed in the sample* | *Difference between sample means* |
| --- | --- | --- | --- |
|  |  |  |  |
| Agric. production (kg) | 3, 143.489 | 4, 294.611 | 1, 151.1211*** |
| Agric. income (USD 2005 PPP) | 1,721.332 | 2,091.942 | 370.61* |
| Cultivated land (ha) | 0.531 | 0.638 | 0.1070 |
| Irrigated land (ha) | 0.3169 | 0.4324 | 0.1156*** |
| Agric. shocks (number) | 14.4011 | 14.3994 | -0.0016 |
| Poverty headcount ratio | 0.2267 | 0.2112 | -0.0155** |
| Gini coefficient | 0.4018 | 0.396 | **-**0.0057 |
| Distance to nearest HPP (km) | 19.5249 | 17.3134 | -2.115*** |
| No. HPP downstream | 0.7343 | 0.864 | 0.1297** |
| No. HPP upstream | 0.8896 | 1.1233 | 0.2337*** |
| Education of household head (years) | 7.6329 | 7.8516 | 0.2187 |
| Female household head (0/1) | 0.2678 | 0.1709 | -0.0968*** |
| Age of household head (years) | 53.545 | 52.7191 | -0.8259 |
| Belonging to ethnic minority (0/1) | 0.1922 | 0.2103 | 0.0181 |
| No. household members on-farm | 1.487 | 1.8422 | 0.3552*** |
| No. household members off-farm | 0.4522 | 0.4143 | -0.0379 |
| Household size | 3.7102 | 4.14 | 0.4294*** |
| ^*^ *p* < 0.1, ^**^ *p* < 0.05, ^***^ *p* < 0.01, Source: Own calculation from TVSEP data | | | |
