# Supplementary material for: Small hydropower plants and livelihoods of the local population in rural Vietnam
Source: PLoS One. 2025 Mar 24;20(3):e0317247. doi: 10.1371/journal.pone.0317247 (PMC11932490; doi:10.1371/journal.pone.0317247)
Supplement: S9 Table — (DOCX) [file pone.0317247.s009.docx]

S 9 Table. HPPs’ effect on household size using household size for equations (1) & (2)

|  | *Distance to nearest HPP* | *Distance to nearest HPP*  *downstream* | *Distance to nearest HPP*  *upstream* |
| --- | --- | --- | --- |
|  |  |  |  |
| *Panel A: Whole sample* |  |  |  |
| Nucleus household size | 0.001 | 0.2 | **-**0.02 |
|  | (0.006) | (0.53) | (0.014) |
| *Panel B: Dak Lak* |  |  |  |
| Nucleus household size | -0.01 | 0.2 | -0.03*** |
|  | (0.01) | (0.53) | (0.008) |
| Standard errors clustered at village level in parentheses, ^*^ *p* < 0.1, ^**^ *p* < 0.05, ^***^ *p* < 0.01, Source: Own calculation from TVSEP data | | | |
