# Supplementary material for: Small hydropower plants and livelihoods of the local population in rural Vietnam
Source: PLoS One. 2025 Mar 24;20(3):e0317247. doi: 10.1371/journal.pone.0317247 (PMC11932490; doi:10.1371/journal.pone.0317247)
Supplement: S10 Table — (DOCX) [file pone.0317247.s010.docx]

S 10 Table. HPPs’ effect on average yield for equations (1) & (2)

|  | *Distance to nearest HPP* | *Distance to nearest HPP* | *Distance to nearest HPP* |
| --- | --- | --- | --- |
|  |  | *downstream* | *upstream* |
| *Panel A: Whole sample* |  |  |  |
| Average yield per hectare | 37.21 | 13,003.2** | 477.3 |
|  | (119.65) | (5,970.3) | (654.6) |
| *Panel B: Dak Lak* |  |  |  |
| Average yield per hectare | -111.7 | 12,942.0 | 21.47 |
|  | (134.7) | (5,975.3) | (211.6) |
| Standard errors clustered at village level in parentheses, ^*^ *p* < 0.1, ^**^ *p* < 0.05, ^***^ *p* < 0.01, Source: Own calculation from TVSEP data | | | |
