# Supplementary material for: Small hydropower plants and livelihoods of the local population in rural Vietnam
Source: PLoS One. 2025 Mar 24;20(3):e0317247. doi: 10.1371/journal.pone.0317247 (PMC11932490; doi:10.1371/journal.pone.0317247)
Supplement: Attachment — Submitted filename: PONE-D-24-38432_R1_(1)_Response_Letter.docx [file pone.0317247.s011.docx]

**Responses to the Editor and Reviewers of PONE-D-24-38432**

**"Small hydropower plants and livelihoods of the local population in rural Vietnam”**

S. Ezhil Vendan, PhD, Academic Editor, PLOS ONE

Hannover, November 14, 2024

Dear Dr. S. Ezhil Vendan,

Thank you very much for allowing us to revise and resubmit our manuscript. We would also like to thank you and the two reviewers for the constructive comments, which helped us to considerably improve the manuscript. We have acknowledged this in our acknowledgements section. As you can see now in this revised version, all of the comments have been carefully considered and addressed. Our detailed responses to your and their comments are included in this letter.

We look forward to your decision regarding the revised manuscript.

Yours sincerely,

**Responses to the Academic Editor (your comments in blue and our responses in black)**

The manuscript organization should strictly adhere to the authors guidelines of the journal. Accordingly, authors should revise the manuscript under the headings of Introduction, Materials and Methods, Results, and Discussion sections only.

Thank you very much for your comment.

We restructured the paper according to your guidelines regarding headings, citations, figures, and tables. However, we decided to keep the Results and Discussion together in one section and add an optional section Conclusion. We feel that disentangling the Results and Discussion would lead to a repetition of findings. Another advantage of keeping it together is that the content is more reader-friendly as we present each of our findings and discuss it at the same time. Nevertheless, if you wish us to disentangle the two sections, we are happy to follow your suggestion.

The introduction is too vague and elaborated. Study background information should be very clear and more specific to the study objectives. Please remove all subheadings, figures, equations and broad literature review in the introduction section. Authors should minimize the introduction section.

Thank you for this comment. We shortened the introduction as much as possible and rephrased it so that background information is clearer and more tightly connected to our objectives. Additionally, we also removed as much as possible of the broad literature review from the introduction. Instead, we decided to add a second section on the literature review and conceptual framework as we have seen it in many articles published in PLOS ONE. In addition, Reviewer 1 asked us to add to the literature review.

Please revise the other sections strictly with the headings “Materials and Methods, Results, and Discussion sections and avoid too general statements.

We revised the manuscript completely and removed too general statements wherever we detected them.

Study conclusions are not clear in the manuscript. Please revise the conclusion section with study highlights and findings significance. Remove elaborated descriptions from the conclusion.

We rephrased the Conclusion completely by removing the main part of the elaborated descriptions and focusing on key findings, study highlights and their implications. Additionally, we added more information on limitations of our study as a scope for future research.

**Responses to Reviewer 1 (your comments in blue and our responses in black)**

We would like to thank you for your constructive comments and suggestions, which helped us to considerably improve the manuscript. We have acknowledged this in our acknowledgements section. As you can see in this revised version, all of your comments have been carefully considered and addressed.

I suggest change the word hydropower in Keywords: Hydropower because is on the title.

Thank you very much for hinting us to this fact. We removed this word.

Use third-person perspective throughout the text to maintain a formal and objective tone.

We adjusted the manuscript accordingly.

The motivation could be strengthened by providing more specific examples or recent data on the impact of HPPs, particularly focusing on small HPPs, to highlight the current relevance of the research.

Thank you very much for your comment. We agree that this would highlight the relevance of the research and added evidence on small HPPs to our literature review. We did not include this in the introduction of the manuscript which motivates the paper since the editor asked us to significantly shorten the introduction of the manuscript.

It would be beneficial to explicitly state how the study's findings could influence policy or practical applications. Additionally, briefly outlining the main findings in the introduction could further emphasize the study's contribution.

Thank you again for helping us improving our introduction. We added the key findings and their implications to the penultimate paragraph of the introduction section.

The description is adequate but could be improved by specifying the exact timeframe covered by the TVSEP dataset. Although you mention the use of data from 2007 to 2017, providing specific dates of data collection, if available, would be beneficial.

We added information on the data collection to Section 2.3. Data.

*“During data collection, enumerators always ask for data from the same reference period (May until April). The data collection of the six waves used in this study were collected in 2007, 2008, 2010, 2013, 2016, and 2017.”*

The description of the MRFI dataset is clear. It would be helpful to mention if the dataset is regularly updated and how current the data are. This will support the temporal validity of your results.

We added information to the manuscript as far as information is available to us. To the best of our knowledge, the data was updated on a regular basis. The latest update we use for the analysis is from 2019 which matches the TVSEP data as we use waves up to 2017 (the TVSEP data in Vietnam are available only until 2017 for all three provinces).

The methodology for merging data using GPS and ArcGIS is well-explained. You might enhance this section by briefly describing how data quality was ensured during the merging process and the accuracy of GPS coordinates. This will bolster confidence in your results.

We added information on the merging process and the accuracy of the GPS coordinates in Section 3.1 Data.

*“Data quality during the merging process was ensured by using the same coordinate system (GCS_WGS_1984). GPS data usually contained information of six digits.”*

In the section about using ArcGIS, you mention that results are transferred to Stata for analysis. Adding details on how data conversion was managed and if there were any significant challenges would be beneficial.

We added information on the process and conversion at the beginning of Section 3.2

*“The ArcGIS and the TVSEP datasets were merged by exporting the ArcGIS analysis results (attribute tables) into Excel and further on reading them into Stata. As not only GPS information for the villages in the TVSEP data were added but also the village identifier, merging the data sets according to village identifier in Stata was possible.”*

The details on agricultural variables are good, but you could add specific examples of how variables like "agricultural shocks" and "expected number of droughts" were measured. Clarifying these points will improve transparency and reproducibility.

We added the information to Section 3.1 Data and Table 1 in the Supplementary Information Section (S1 Table).

When calculating poverty indices and Gini coefficients, it would be helpful to provide more information on the poverty threshold used and the definition. This will contextualize the results.

We use a threshold of 1.90 USD converted to 2005PPP. The information can also be found in our manuscript in Section 3.1.

*“Furthermore, with respect to poverty and inequality, data from the TVSEP project was used to calculate village-wise poverty headcount ratios using the poverty line of 1.90 USD 2005 PPP and Gini coefficients using households annual total net income (in 2005 USD PPP).”*

The tables provide a useful overview, but a more detailed analysis of observed trends would be beneficial. For instance, explaining why changes in cultivated land area and agricultural income are significant or not can offer deeper insights into the results.

We added and elaborated on the explanation to the description of the results from Table 2.

*“A possible explanation for this result is that most of the land suitable for agriculture has already been converted into cropland, while farming households more and more use irrigation to better control and increase agricultural yield.”*

The explanation of regression models is clear but could be improved by detailing how control variables were selected and why they are relevant to the study. Additionally, briefly explaining why fixed effects models were chosen and how they help control for unobserved variables would add rigor.

An explanation how and why independent variables were chosen is added to the methodology section, along with more information on fixed effects.

*“Fixed effects are preferably used for this analysis, first, because the Hausman Test indicated that fixed effects produce more reliable estimates, and second to control for time-varying factors in the whole sample and for each province individually. Thus, year fixed effects and year-province fixed effects are included. Province fixed effects help to control for structural differences between the three provinces in the TVSEP project. Furthermore, household fixed effects are also considered to account for the underlying and unobservable trends and characteristics of each household.”*

*“The other independent variables included in the regressions are represented by the vector* $X_{ist}^{'}$*. It includes education, gender and age of the household head, the number of household members engaged in agriculture and in off-farm labor, and whether the household belongs to an ethnic minority. These variables were chosen due to previous studies where these variables had significant effects on agricultural outcomes such as Nguyen et al. [42].”*

Providing justification for using cluster-robust standard errors at the village level would also strengthen the methodology.

Explanation is added to the methodology section.

*“Standard errors are clustered at the village level because the distance analysis in ArcGIS is based on village level and, thus, the treatment due to HPPs affects each household in the village.”*

Clearly explain why results differ between the overall sample and Dak Lak.

The explanation at the beginning of chapter 3 was elaborated in more detail.

*“As Dak Lak has by far the highest density of HPPs (see Table 1), Dak Lak might be more suitable for the construction of HPPs due to its mountainous landscape. Furthermore, Dak Lak has soil characteristics which are more suitable for cultivating water-intensive crops such as coffee and therefore has a higher need for regular water provision. This might lead to different results for the impact of HPPs and, therefore, the analysis is first conducted for the whole sample and afterwards for the restricted sample including only data from Dak Lak.”* Provide more context on how socio-economic variables like education, gender, and age of household heads influence outcomes related to HPPs.

Thank you very much for your comment. We very much hope we understood your concerns correctly The HPPs in the sample are publicly owned and not owned by the interviewed households which can be checked within the asset section of the TVSEP data and also within the HPP data. Therefore, these variables should have no effect on the placement of HPPs.

Compare findings more thoroughly with previous studies, such as Duflo and Pande (2007), highlighting both similarities and differences.

We added some more comparison to Section 4.1 and also discussed some differences below Table 3. The differences are mainly the reason why we would restrain from an estimate-by-estimate comparison.

*“The findings are in line with the results of Duflo and Pande [5] as they also report no effects on poverty and equality indicators. Additionally, they also consider increased vulnerability to rainfall shocks to be likely as access is limited, especially in years with insufficient rainfall [21]. A further comparison with Duflo and Pande [5] reveals that the estimates for the irrigated area are similar to the results in Table 4 (0.33% vs. 0.5%). However, while Duflo and Pande [5] look at outcomes at district level, the study at hand looks at outcomes at household or village level. Additionally, this study uses the distance to the closest HPP, while Duflo and Pande [5] use the number of HPPs in the district. Instead of using agricultural yield as independent variable as in Duflo and Pande [5] this study uses agricultural income because aggregating yields from different types of crops seemed unreliable.”*

Discuss why some results are significant in one province but not in others, and the possible reasons for insignificant results.

After table 5 at the very end of Section 4.1 we added some more elaborated explanation on why we might see insignificant results in Dak Lak compared to the results for the whole sample.

*“While Dak Lak is a highland and landlocked province with a higher share of ethnic minorities, the other two provinces are coastal provinces. Duflo and Pande [5] argue that the results in districts where HPPs are located (upstream districts) tend to be noisy and insignificant. Since Dak Lak has the highest HPP density, the insignificant results might be due to this noise. Another explanation for the insignificant results in Dak Lak could be the already high density of HPPs before 2007 so that adding more HPPs, even though the HPPs are closer together, might not have any effect. Consistent with the hypotheses, positive effects on agricultural outcomes emerge when small HPPs are located nearby, while the impacts of large HPPs are more diverse [5].”*

Expand on the robustness of the results by detailing how robustness checks address potential endogeneity issues and other concerns.

We included more explanation what we did with the separate robustness checks at the beginning of the subsection and also by adding information to every single robustness check performed.

*“Several robustness checks were performed in order to test the results for possible endogeneity issues. The first check investigates the fact that HPPs require certain geographical characteristics such as a river and certain gradients of the surrounding area [5]. This is followed by several robustness checks that test the robustness of the results against different definitions of dependent and independent variables. Last, the results are compared with voluntary migration and resulting selection bias.”*

Analyze in more detail how HPPs impact upstream and downstream populations differently, including specific examples or case studies.

We added further evidence to highlight the differences between upstream and downstream populations using case studies.

*“Unfortunately, there are few studies that investigate non-resettled communities in terms of farming outcomes, distinguishing between upstream and downstream locations. However, the results of the Bui dam project in Ghana indicate that most farmers interviewed were not affected by the construction [43]. Still, some report land shortages because upstream households tend to migrate to downstream areas. This result supports the findings discussed in this chapter as it indicates that downstream farming is more profitable than upstream.”*

Improve the visual presentation of data, such as using clearer graphs or tables, and ensure they are well-labeled and easy to interpret.

We adjusted the labels and explanations in the tables to improve labelling and make interpreting easier.

Discuss selection bias more explicitly, including how it was addressed and its potential impact on the results.

We added some information about our concerns regarding selection bias and tried to explain in more detail how we addressed it and what this means for our results.

*“Due to the construction of HPPs, disadvantaged households might migrate and thus drop out of future data collection for the TVSEP project. There is a concern that this introduces selection bias as households negatively affected by HPP construction choose to migrate and the dataset only includes households that benefit or are not affected by HPPs. Therefore, a balanced sample is created and compared with the sample of households for which data for all six waves are not available. To test for differences between these two samples, t-tests are calculated. S 8 Table shows significant differences between the two groups and reveals that households that leave the sample are worse off than households that remain in the area. For example, such households are more likely to have a female household head, are poorer, irrigate smaller parts of their cropland, have less agricultural production, and have fewer household members engaged in farming. However, the unbalanced sample is much smaller than the balanced sample. Additionally, they are surrounded by fewer upstream and downstream HPPs and are generally farther away from the nearest HPP. To further test for selection bias, the nucleus household size is added as a dependent variable to test whether migration increases with the construction of HPPs (S 9 Table). The results for Dak Lak show that HPPs have no impact on the size of nucleus households for the upstream population but the opposite for downstream households. One reason for this can be the generally positive effect of HPPs on the population downstream. In conclusion, distinguishing between households remaining in the sample and households leaving the sample reveals structural differences between the two groups. However, it is not clear whether this is due to the construction of HPPs or other household-specific characteristics. The structure of the present dataset does not allow for further investigation and therefore this relationship deserves detailed analysis and opens future research opportunities.”*

Identify specific areas for future research based on current findings and limitations, and suggest additional methodological approaches.

Areas for future research based on current findings and limitations along with further methodological approaches are presented and discussed in the conclusion.

*“Previous research revealed that the construction of large HPPs negatively affects soil fertility, increases water salinity and negatively impacts fish populations. This opens further research opportunities. First, analyzing the impact of small HPPs on the environment alone. Second, by taking these environmental impacts into account when investigating impacts on agricultural outcomes or general well-being. Interdisciplinary research, for example involving natural sciences, could help to draw an overall picture of this connections. Furthermore, future research should also focus on the relationship between HPPs and migration. While forced displacement due to HPPs is well researched [16,21,44], it is still unclear whether migration flows change in the presence of HPPs. On the one hand, people might move closer to HPPs because they expect promising benefits such as job opportunities [5]. On the other hand, people out-migrate because they experience or expect negative impacts from nearby HPPs. The robustness checks suggest that there may be a significant difference between households that out-migrate and those that stay nearby, leaving room for further scientific investigation as this introduces a selection bias in the dataset. One way to deal with this problem is to combine the TVSEP data with the TVSEP migration data, where the project aims to track households and household members who have moved to other areas and are therefore missing in the original TVSEP data set after they migrated. Additionally, the descriptive results reveal that the distance between HPPs and villages is still substantial at approximately 10km which might influence the results. Consequently, the aim of identifying villages and households close to HPPs and then collecting targeted data on these households that would allow comparison with villages further away from HPPs would be an area for future research.”*

Clearly articulate how the findings on small HPPs compare with those on large HPPs, beyond noting that small HPPs show no significant negative effects on upstream populations.

We added on this by comparing the results again with those of Duflo and Pande (2007).

*“While Duflo and Pande [5] investigate the effects of large HPPs, this analysis focuses on small HPPs. The results suggest that small HPPs increase the share of irrigated area for downstream households but not for upstream households, while Duflo and Pande [5] find an increase in absolute irrigated area for large HPPs for both populations. While this can be attributed to the smaller size of the HPPs, the results reveal no negative impacts on the upstream population that Duflo and Pande [5] report for large HPPs. Therefore, the lack of a significant increase in irrigated land area for small HPPs is outweighed by other negative effects for large HPPs which are not present for small HPPs, e.g. relocation or lower agricultural yields. Another advantage of small HPPs is that fewer households need to be relocated and the negative impacts on land quality and aquatic life might not be as severe compared to large HPPs. However, the connection with environmental effects is the subject for future research and requires inter-disciplinary data collection and analysis.”*

Provide a more detailed explanation of the specific environmental impacts of small HPPs and compare these impacts with those of large HPPs.

As far as we know the literature on environmental effects did not distinguish between effects from large and small HPPs. We added environmental effects to the conclusion section and explained how they could be addressed in an inter-disciplinary way which also includes economics

*“Previous research revealed that the construction of large HPPs negatively affects soil fertility, increases water salinity and negatively affects fish populations. This opens further research opportunities. First, analyzing the impact of small HPPs on the environment alone. Second, by taking these environmental impacts into account when investigating impacts on agricultural outcomes or general well-being. Interdisciplinary research, for example involving natural sciences, could help to draw an overall picture of this connection.”*

Discuss the implications of the observed positive effects of small HPPs on downstream populations in the context of their overall benefit compared to large HPPs.

Elaborate on how the results might influence policy recommendations for promoting small HPPs, including specific measures to mitigate potential negative impacts.

We added on the comparison of the effects of small HPPs against large HPPs and also added some more implications for policymakers in the conclusion.

*“This implies that small HPPs are at least as beneficial as large HPPs and should therefore also be promoted. The results suggest that small HPPs have similar positive effects on agriculture as large HPPs, but the negative effects, for example on the upstream population, might be smaller. Therefore, policymakers should promote the construction of small HPPs and not focus on large HPPs. However, there is scope for future research into the effect of multiple small HPPs in close proximity to the same river on the well-being of neighboring populations. Besides, policymakers should be aware of the diverging effects for upstream and downstream populations to prevent social conflicts through appropriate and sufficient compensation as well as transparent communication and the involvement of affected villages in the planning process.”*

Address the gap in understanding regarding the relationship between HPPs and migration, and suggest how future research could explore this aspect in more detail.

We added a possible solution to this problem and explained the issue in more detail in the robustness checking section.

*“Furthermore, future research should also focus on the relationship between HPPs and migration. While forced displacement due to HPPs is well researched [16,21,44], it is still unclear whether migration flows change in the presence of HPPs. On the one hand, people might move closer to HPPs because they expect promising benefits such as job opportunities [5]. On the other hand, people out-migrate because they experience or expect negative impacts from nearby HPPs. The robustness checks suggest that there may be a significant difference between households that out-migrate and those that stay nearby, leaving room for further scientific investigation as this introduces a selection bias in the dataset. One way to solve this problem is to combine the TVSEP data with the TVSEP migration data, where the project aims to track households and household members who have moved to other areas and are therefore missing in the original TVSEP dataset after they migrated.”*

Highlight any limitations of the current study and how they might affect the interpretation of results, including the scope of the panel dataset and potential biases.

We added some more limitations of this study to the end of the conclusion.

*“Additionally, the descriptive results reveal that the distance between HPPs and villages is still substantial at about 10 km, which might influence the results. Consequently, the aim of identifying villages and households close to HPPs and then collecting targeted data on these households that would allow comparison with villages further away from HPPs would be an area for future research.”*

**Responses to Reviewer 2 (your comments in blue and our responses in black)**

We would like to thank you for your constructive comments, which helped us to considerably improve the manuscript. We have acknowledged this in our acknowledgements section. As you can see now in this revised version, all of your comments have been carefully considered and addressed.

The introduction lacks a clear, concise topic sentence for each paragraph, leading to unclear transitions between thoughts. The authors should introduce key ideas more effectively to improve coherence.

Thank you. We restructured and completely re-phrased the introduction to make it more stringent and also address the comments from the other reviewer and editor. The first paragraph gives background information on why studying the impact of HPPs on agriculture and poverty is relevant and interesting. The second paragraph identifies research gaps and objectives of the study. The third paragraph describes how these objectives will be addressed, while the fourth paragraph briefly summarizes key findings and conclusions. Finally, the last paragraph outlines the structure of the study.

The introduction does not include hypotheses or research questions, which are crucial in guiding the reader through the study's aim. The authors should include these to clarify the direction of the research-

We included hypotheses and clear aims to the introduction. The part now reads as followed:

*“…Thus, the first aim of this study is to identify the effect of small HPPs on farming households in rural Vietnam, hypothesizing that agriculture can benefit from a more stable irrigation. This in turn might lead to a reduction in poverty in these communities. Furthermore, the analysis of the effect of HPPs relied primarily on case studies rather than large panel or cross-sectional data [15]. Therefore, the second aim of this study is to analyze the effect of small HPPs on the livelihoods of non-relocated rural Vietnamese households using a large panel dataset.”*

The introduction lacks a detailed literature review that situates the study within the existing body of research. Including a review of relevant literature would provide context and justify the need for this research.

Thank you for your comment. The editor asked us to shorten the introduction section. Therefore, we are not able to provide a more detailed literature review in this section. We, therefore, review the relevant literature in Section 2.1.

The introduction does not provide information about the current state of agriculture in Vitamin.

We added information on the Vietnamese agricultural sector to the introduction.

*“… This is particularly relevant for Vietnam as 38% of Vietnam’s workforce was still engaged in agriculture in 2018 and around 16.3% of gross domestic product (GDP) in 2018 was generated by the agricultural sector [15] …”*

The current Research Design sub-section does not explain the design of this research. Instead, the current Section 2.2 contains Data Collection (please rename it). Therefore, the authors should make a Research Design sub-section to explain the step-by-step (stages) of this research. It is critical to convince readers that this research was conducted systematically and included necessary research activities. For each stage, please explain its objective(s), technique/approach(es) being used, and outcome(s) expected from the stage.

In the submitted manuscript, Section 2.1 contains the literature review while the data collection process is described in Section 3.1. The data was collected by a team from the TVSEP research project “Poverty dynamics and sustainable development: A long-term panel project in Thailand and Vietnam". Building on previous work by the research unit FOR 756 of the Deutsche Forschungsgemeinschaft (DFG), this project aims at providing a long-term panel. It aims to cover 4,400 households, 2,200 households in each country. The study sites in Vietnam cover the rural provinces of Dak Lak, Ha Tinh, and Thua Thien Hue, and in Thailand the three rural provinces of Buriram, Ubon Ratchathani and Nakhon Phanom. As the aim of the project is to study poverty and development, provinces were selected based on poverty indicators such as low average per capita income, poor infrastructure or a high dependence on agriculture. The sampling within the provinces ensures a representative sample of the rural population and was done in three steps (see Hardeweg, Bernd, Stephan Klasen, and Hermann Waibel. 2013. “Establishing a Database for Vulnerability Assessment.” In: Vulnerability to Poverty: Theory, Measurement and Determinants, with Case Studies from Thailand and Vietnam, edited by Stephan Klasen and Hermann Waibel, 50–79. London: Palgrave Macmillan UK): First, a stratification strategy was applied to account for different population densities between sub-districts. Second, two villages per sub-district were randomly selected with a probability proportional to the population of the respective sub-district. Third, ten households were randomly selected from each village and information was collected on their socio-demographic characteristics, sources of income, financial situation, land and asset endowment, and self-reported environmental shocks. Additionally, we describe in this section how the data sets were merged and necessary variables constructed. Section 3.3 (now 2.4) explains the econometric approach used to investigate the research questions. We added some more information to this section to make it easier to follow our idea.

Results and Discussion do not get along. The authors should focus on presenting the results of this study in this Results section. The authors do not compare their findings with those of other studies in similar or different contexts. A comparative discussion, especially with findings from studies in different regions or farming systems, would provide a more comprehensive understanding of how generalizable or context-specific the results are.

We decided to keep the combination of Results and Discussion as we feel that disentangling these two sections would lead to a repetition of findings and is not as reader friendly as is a direct discussion of the results after they have been presented. We added comparisons with other studies to the section.

*“A further comparison with Duflo and Pande [5] reveals that the estimates for the irrigated area are similar to the results in Table 4 (0.33% vs. 0.5%). However, while Duflo and Pande [5] look at outcomes at the district level, our study looks at outcomes at the household or village level. Additionally, our study uses the distance to the closest HPP, while Duflo and Pande [5] use the number of HPPs in the district. Instead of using agricultural yield as independent variable as in Duflo and Pande [5] this study uses agricultural income because aggregating yields from different types of crops seemed unreliable.”*

*“Unfortunately, there are few studies that investigate non-resettled communities in terms of farming outcomes, distinguishing between upstream and downstream locations. However, the results of the Bui dam project in Ghana indicate that most farmers interviewed were not affected by the construction [43]. Still, some report land shortages because upstream households tend to migrate to downstream areas. This result supports the findings discussed in this chapter as it indicates that downstream HPP farming is more profitable than upstream.”*

The current form of Conclusion mixes up the summary and key findings of this study with suggestions arising from the findings. The authors may simply consider using 3 paragraphs:

i. Summary and key findings of this study
ii. Managerial and policy implications arising for each key finding
iii. Insights for future research arising from the key findings and limitations of this research

We rephrased and restructured the conclusion according to your suggestions, which was very helpful in making the conclusion more stringent.

**Responses to Journal requirements (your comments in blue and our responses in black)**

1. Please ensure that your manuscript meets PLOS ONE's style requirements, including those for file naming. The PLOS ONE style templates can be found at

https://journals.plos.org/plosone/s/file?id=wjVg/PLOSOne_formatting_sample_main_body.pdf and

https://journals.plos.org/plosone/s/file?id=ba62/PLOSOne_formatting_sample_title_authors_affiliations.pdf

We downloaded the referred files and adjusted the manuscript and other material accordingly.

2. Thank you for stating the following financial disclosure:

“German Research Foundation”

Please state what role the funders took in the study.  If the funders had no role, please state: "The funders had no role in study design, data collection and analysis, decision to publish, or preparation of the manuscript."

If this statement is not correct you must amend it as needed.

Please include this amended Role of Funder statement in your cover letter; we will change the online submission form on your behalf.

Our study was not funded by the German Research Foundation. This refers to the TVSEP project which granted us access to the collected data. We adjusted the acknowledgements accordingly and removed the funding information.

3. Thank you for stating the following in the Acknowledgments Section of your manuscript:

“This study relies on data from the long-term project No. 20220831434900116103, funded by the Deutsche Forschungsgemeinschaft (DFG). For more detailed information, see http://www.tvsep.de”

We note that you have provided funding information that is currently declared in your Funding Statement. However, funding information should not appear in the Acknowledgments section or other areas of your manuscript. We will only publish funding information present in the Funding Statement section of the online submission form.

Please remove any funding-related text from the manuscript and let us know how you would like to update your Funding Statement. Currently, your Funding Statement reads as follows:

“German Research Foundation”

Please include your amended statements within your cover letter; we will change the online submission form on your behalf.

Our study was not funded by the German Research Foundation. This refers to the TVSEP project which granted us access to the collected data. We adjusted the acknowledgements accordingly and removed the funding information.

4. When completing the data availability statement of the submission form, you indicated that you will make your data available on acceptance. We strongly recommend all authors decide on a data sharing plan before acceptance, as the process can be lengthy and hold up publication timelines. Please note that, though access restrictions are acceptable now, your entire data will need to be made freely accessible if your manuscript is accepted for publication. This policy applies to all data except where public deposition would breach compliance with the protocol approved by your research ethics board. If you are unable to adhere to our open data policy, please kindly revise your statement to explain your reasoning and we will seek the editor's input on an exemption. Please be assured that, once you have provided your new statement, the assessment of your exemption will not hold up the peer review process.

Thank you for the information.

5. We note that Figures to 2,3 and 4 in your submission contain [map/satellite] images which may be copyrighted. All PLOS content is published under the Creative Commons Attribution License (CC BY 4.0), which means that the manuscript, images, and Supporting Information files will be freely available online, and any third party is permitted to access, download, copy, distribute, and use these materials in any way, even commercially, with proper attribution. For these reasons, we cannot publish previously copyrighted maps or satellite images created using proprietary data, such as Google software (Google Maps, Street View, and Earth). For more information, see our copyright guidelines: http://journals.plos.org/plosone/s/licenses-and-copyright.

We require you to either (1) present written permission from the copyright holder to publish these figures specifically under the CC BY 4.0 license, or (2) remove the figures from your submission:

a. You may seek permission from the original copyright holder of Figures to 2,3 and 4 to publish the content specifically under the CC BY 4.0 license.

We recommend that you contact the original copyright holder with the Content Permission Form (http://journals.plos.org/plosone/s/file?id=7c09/content-permission-form.pdf) and the following text:

“I request permission for the open-access journal PLOS ONE to publish XXX under the Creative Commons Attribution License (CCAL) CC BY 4.0 (http://creativecommons.org/licenses/by/4.0/). Please be aware that this license allows unrestricted use and distribution, even commercially, by third parties. Please reply and provide explicit written permission to publish XXX under a CC BY license and complete the attached form.”

Please upload the completed Content Permission Form or other proof of granted permissions as an ""Other"" file with your submission.

In the figure caption of the copyrighted figure, please include the following text: “Reprinted from [ref] under a CC BY license, with permission from [name of publisher], original copyright [original copyright year].”

b. If you are unable to obtain permission from the original copyright holder to publish these figures under the CC BY 4.0 license or if the copyright holder’s requirements are incompatible with the CC BY 4.0 license, please either i) remove the figure or ii) supply a replacement figure that complies with the CC BY 4.0 license. Please check copyright information on all replacement figures and update the figure caption with source information. If applicable, please specify in the figure caption text when a figure is similar but not identical to the original image and is therefore for illustrative purposes only.

The following resources for replacing copyrighted map figures may be helpful:

USGS National Map Viewer (public domain): http://viewer.nationalmap.gov/viewer/

The Gateway to Astronaut Photography of Earth (public domain): http://eol.jsc.nasa.gov/sseop/clickmap/

Maps at the CIA (public domain): https://www.cia.gov/library/publications/the-world-factbook/index.html and https://www.cia.gov/library/publications/cia-maps-publications/index.html

NASA Earth Observatory (public domain): http://earthobservatory.nasa.gov/

Landsat: http://landsat.visibleearth.nasa.gov/

USGS EROS (Earth Resources Observatory and Science (EROS) Center) (public domain): http://eros.usgs.gov/#

Natural Earth (public domain): http://www.naturalearthdata.com/

Thank you for the information. The satellite images are open source. We upload a proof of granted permissions as an ""Other"" file with your submission.
